# Beta-Catenin Is Vital for the Integrity of Mouse Embryonic Stem Cells

**DOI:** 10.1371/journal.pone.0086691

**Published:** 2014-01-21

**Authors:** Angelo Raggioli, Dirk Junghans, Stefan Rudloff, Rolf Kemler

**Affiliations:** 1 Max-Planck Institute of Immunobiology and Epigenetics, Emeritus Laboratory, Freiburg, Germany; 2 Institute of Embryology and Stem Cell Biology, Department of Biomedicine, University of Basel, Basel, Switzerland; Sanford Burnham Medical Research Institute, United States of America

## Abstract

β-catenin mediated Wnt-signaling is assumed to play a major function in embryonic stem cells in maintaining their stem cell character and the exit from this unique trait. The complexity of β-catenin action and conflicting results on the role of β-catenin in maintaining the pluripotent state have made it difficult to understand its precise cellular and molecular functions. To attempt this issue we have generated new genetically modified mouse embryonic stem cell lines allowing for the deletion of β-catenin in a controlled manner by taking advantage of the Cre-ER-T2 system and analyzed the effects in a narrow time window shortly after ablation. By using this approach, rather then taking long term cultured β-catenin null cell lines we demonstrate that β-catenin is dispensable for the maintenance of pluripotency associated genes. In addition we observed that the removal of β-catenin leads to a strong increase of cell death, the appearance of multiple clustered functional centrosomes most likely due to a mis-regulation of the polo-like-kinase 2 and furthermore, alterations in chromosome segregation. Our study demonstrates the importance of β-catenin in maintaining correct cellular functions and helps to understand its role in embryonic stem cells.

## Introduction

Mouse embryonic stem cells (ES cells) are isolated from the inner cell mass of pre-implantation embryos at blastocyst stage and exhibit the two characteristics defining embryonic stem cells, which are prolonged self-renewal properties and the ability to differentiate into all three germ-layers – so called pluripotency [1], [2]. Understanding the molecular and cellular mechanisms that allow these cells to maintain their characteristics is subject of extensive research already for decades. Among the many intrinsic and extrinsic signaling pathways that have been identified so far [3], [4] the role of the Wnt/β-catenin signaling in maintaining pluripotency remained for a long time mystic not least because of contradictory findings. Beside its function in mediating cell adhesion by bridging classical cadherins with the cytoskeleton β-catenin is known for its essential role as intracellular mediator of the canonical Wnt-signaling pathway [5], [6], [7], [8]. However, it appears that the key pluripotency genes of mouse ES cells Nanog, Oct4 and Sox2 are directly or indirectly regulated in a context specific manner by β-catenin that involves the transcription factors TCF1 and TCF3 (excellently reviewed by [9], [10], [11] and [12], [13]).

Chemical inhibition of GSK3β or short-term treatment with soluble Wnt3a provided the initial evidence for an important role of Wnt/β-catenin signaling in maintaining pluripotency [14], [15], [16]. However, several other studies reported conflicting or inconsistent results regarding the role of Wnt/β-catenin in maintaining the pluripotency state [17], [18], [19], [20], [21]. For example long-term treatment with Wnt3a results in differentiation of mouse ES cells into mesendodermal lineage [22], [23] whereas Wnts have been shown in vivo and in vitro to prevent differentiation of ES cells into epiblast cells and furthermore, facilitate derivation and establishment of ES cell lines [24]. Interestingly, β-catenin-null embryos exhibit normal development until early gastrulation [25], [26]. Several Wnt/β-catenin mutant ES cell lines have been analyzed by different groups to elucidate the role of β-catenin in mouse ES cells. Their partially conflicting results on the role of β-catenin in ES cells might not only be a result of strain, origin or culturing differences but also due to adaption and compensatory mechanisms [17], [19], [20]. For example it was found that β-catenin-null ES cells can up-regulate plakoglobin that might compensate at least partially for the adhesion function of β-catenin [12], [26], [27]. Most studies in the past analyzing the function of β-catenin in ES cells relied on β-catenin ablated ES cells, which were cultured and passaged over a longer period. In this study, we have analyzed in detail the early cellular responses of ES cells at early time-points after genetic ablation of β-catenin in order to avoid adaptation of the ES cell by compensatory mechanisms. To control for the temporal loss of β-catenin we have generated new ES cell lines. First, we generated a *βcat^del/flox^* -line (hereafter referred to as SR1 line). Second, a Cre-ER-T2 expression cassette was introduce into this line and stable clones isolated (*βcat^del/flox^ :Cre-ER-T2*, hereafter referred to as ARβ1 line ). By introducing the Cre-ER-T2 system we were able to genetically ablate β-catenin at a defined time-point by adding tamoxifen into the culture medium [28]. By using a combined approach of cell culture and immunocytochemical techniques, gene array analysis, quantitative RT-PCR we analyzed the cellular response of mouse ES cells within the first three days after ablation of β-catenin. We could show that ablation of β-catenin does not lead to a loss or alterations in expression of pluripotency markers. Instead we observed that ES cells underwent apoptosis after removal of β-catenin. We could show that this phenotype is p53 independent and could identify genes as being up-regulated after β-catenin ablation that have been described previously for having pro-apoptotic effects. Importantly, we provide evidence that ablation of β-catenin leads to the appearance of ectopic multiple centrosomes and a mis-function in chromosome separation during cell division.

Our study provides insights into the early cellular events after ablation of β-catenin and helps to understand its function in embryonic stem cells.

## Materials and Methods

### Ethics Statement

Experiments were performed in agreement with the German law on the use of laboratory animals as well as biosafety (S1) and institutional guidelines of the Max Planck society. The use of animals was approved by “Regierungspräsidium Freiburg (Freiburg regional council)” and the animal welfare office of the Max Planck Institue of Immunobiology and Epigenetics, Freiburg, Germany (KE-2iTO-6).

### Cell Culture

ES cells were cultured at 37°C with 5% CO_2_ on irradiated mouse embryonic fibroblasts feeder cells or on 0.1% gelatin (G2500, Sigma) in Dulbecco’s Modified Eagle Medium (DMEM) (41966, Gibco-BRL), supplemented with 15% fetal bovine serum (FBS) (P30-3602, PAA Laboratories), 1× non-essential amino acids (11140, Gibco-BRL), 1x L-glutamine (25030, Gibco-BRL), 1× PenStrep (15070, Gibco-BRL), 1× Sodium pyruvate (11360, Gibco-PRL), 0.001% β-mercaptoethanol (M-7522, Sigma) and 1000 U/ml LIF (Gibco-BRL). 4-hydoxy-tamoxifen (4-OHT) (H-7904, Sigma) was reconstituted in 70% ethanol to a final concentration of 1 mg/ml. For induction of recombination a single dose of 4-OHT was added to the culturing medium with 0.1 µg/ml final concentration. Cells were subsequently analyzed and used for further experiments at day one, two or three. Recombinant Wnt3a (R&D systems) was reconstituted (PBS, 0.1% BSA) with 40 µg/ml and stored at -20°C. Wnt3a was administrated to a final concentration of 100 ng/ml 4.5 hours before harvesting the cells for RNA extraction. siRNAs (Control siRNA, Dharmacon and Plk2: SI01381884, Qiagen) were transfected using Lipofectamine RNAiMAX Reagent (13778, Invitrogen) according the manufacturers protocol.

### Generation of Mouse ES Cell Lines

*βcat* ^del/flox^ mouse ES cells (SR1 cells, Ph.D. thesis Stefan Rudloff, University of Freiburg, 2010) containing one null allele and one allele with exons 2–6 flanked by *loxP* sites [29] were derived from *βcat* ^del/flox^ embryos at E3.5 blastocyst stage using standard protocols and LIF containing medium [30], [31]. A Cre-ER-T2 cassette was cloned into a pCAGGS-IRES-Puro vector and *βcat* ^del/flox^ ES cells transfected with the pCAGGS-Cre-ER-T2-IRES-Puro vector. A stable clonal line was isolated and expanded (*βcat* ^del/flox^ :*Cre-ER-T2* called ARβ1). Treatment of these cells with 4-OHT leads to the generation of *βcat* ^del/del^ alleles.

Wild-type W4 ES cells [32] were used to establish a stably transfected cell line using the pTRIPz vector system (Thermo Scientific). This vector uses the Tet-on/off system to induce expression of sh-RNAs that allows the down regulation of a target gene of interest. We incorporated a *β-catenin* sh-RNA sequence that allowed us upon doxycycline administration to down-regulate β-catenin. The following *β-catenin*–targeting sequence was used: 5′AAAGTATTCACCCACACTG 3′.

### Quantitative Real-time PCR

Total RNA was extracted using TRI Reagent (T9424, Sigma). Subsequently, 1 µg of RNA was reverse transcribed using First strand cDNA Synthesis Kit (K1612, Fermentas) and 5–10 ng cDNA used to perform quantitative RT-PCR using ABsolute QPCR ROX Mix (AB-1139/A, Thermo Scientific) on a ABI PRISM 7300 real time instrument according the manufacturers protocols. Results were obtained from at least three experiments performed in triplicates using the ΔΔCT method.

The following primers were used: *Ddit4l* (fw-ctggtggtctccccacac, bw-tcattgcagtaagaggcacact), *Dffb* (fw-actccagaaggatggttctcc, bw-ggagtgcttggaaagacagc), *Perp* (fw-gaccccagatgcttgttttc, bw-accagggagatgatctggaa), *Plk1* (fw-ttgtagttttggagctctgtcg, bw-cagtgccttcctcctcttgt), *Plk2* (fw-catcaccaccattcccact, bw-tcgtaacactttgcaaatcca), *Plk3* (fw-ggctggcagctcgattag, bw-gttgggagtgccacagatg), *Plk4* (fw-gaaaccaaaaaggctgtgg, bw-ccttcagacgcacactctctc). All other primer pairs can be found in [33].

### Cell Lysate Preparation and Western-blotting

Cells were rinsed PBS (2x, RT), scraped, transferred into 1.5 ml reaction tubes and resuspended in ice-cold RIPA lysis buffer (150 mM NaCl, 1% NP40, 0.5% Sodium Deoxycholate, 0.1% Sodium Dodecyl Sulfate, 50 mM Tris pH 8.0) containing 1 mM EDTA, 0.2 mM Dithiothreitol DTT, 50 mM NaF, 2 mM Na_3_VO_4_ and 1 Complete Protease Inhibitor Cocktail (Roche). The cell lysate was incubated (30′, 4°C) and finally centrifuged (14000 rpm, 10′, 4°C). The supernatant was retained, quantified using BCA (Thermo Scientific) and subsequent analyzed by SDS-PAGE and Western-blotting according standard protocols.

### Immunofluorescence Staining

Cells were plated on gelatin-coated glass cover slips. Cells were rinsed twice with PBS and fixed with ice-cold methanol for 5′ at -20°C. After washing (3×5′, PBST) the cells were blocked for 1 h (10% FBS, 0.1% Tween 20, PBS). Next, cells were incubated with primary antibodies for 1 h in blocking buffer. After washing (3×10′, PBST) the cells were incubated with the appropriate secondary antibody in blocking buffer. Finally, cells were washed (3×10′, PBST) and mounted (DABCO, containing DAPI). Fluorescent images were collected with a fluorescence microscope (Observer Z1, Zeiss and CSU-X1 spinning disk technology, Yokogawa).

### Antibodies

Acetyl-p53 (1∶1000 (WB), 1∶200 (IF), Cell signaling, 2525), Centrin (1∶200, Abcam 11257), E-Cadherin (1∶200, BD, 610101), GAP-DH (1∶50000, Calbiochem, CB1001), Nanog (1∶1000 (WB), 1∶200 (IF), Rolf Kemler, [34]), Oct3/4 (1∶200, Santa Cruz, sc-5279), p53 (1∶1000 (WB), 1∶200 (IF), Cell signaling, 9282), Pericentrin (1∶200, Covance, PRB-432C), Plakoglobin (1∶200, BD, 610253), beta-Actin (1∶1000, Cedarlane, CLT9001), beta-Catenin (1∶1000 (WB), 1∶200 (IF), BD, 610154), beta-Catenin (1∶1000 (WB), 1∶200 (IF), Cell Signaling, 9562). Alexa-Fluorochrome coupled secondary antibodies were used for immunofluorescence and FACS (Invitrogen).

### Nocodazole Washout

Cells were first incubated for 1 h at 4°C in a humid chamber in normal culture medium followed by an incubation for 1 h in a cell culture incubator with nocodazole (50 ng/ml) containing medium (37°, 5% CO_2_)_._ Theafter, cells were washed by three quick rinses with PBS (RT) before addition of nocodazole-free medium (RT). Subsequent, every minute (10 minutes total) one dish was fixed with cold methanol to allow for the analysis of a time series.

### TUNEL Analysis

TdT-mediated dUTP-X nick end labeling (TUNEL) was performed using the In Situ Cell Death Detection Kit, TMR Red (Roche) according to manufacturer’s instruction. Quantification was performed by standard FACS protocols. The experiment was performed four times.

## Results

### Characterization of the ARβ1 and SR1 ES Cell Line

*βcat* ^del/flox^ ES cells (hereafter referred to as SR1 line) were transfected with a vector carrying a Cre-ER-T2 cassette under the control of a chicken beta-actin promoter. By taking advantage of the vector based pyromycin selection cassette a clonal ES cell line was established (*βcat* ^del/flox^ :*Cre-ER-T2*, hereafter referred to as ARβ1). ARβ1 cells do form compact colonies and are virtually indistinguishable from SR1 cells in respect to colony size, compactness, growth rate und cell shape. Administration of 100 ng/ml 4-hydroxy-tamoxifen (4-OHT) to ARβ1 cells was sufficient to achieve efficient recombination of the *β-catenin* locus resulting in ES cells exhibiting genetic ablation of *β-catenin* (*βcat* ^del/del^).

**Figure 1:**
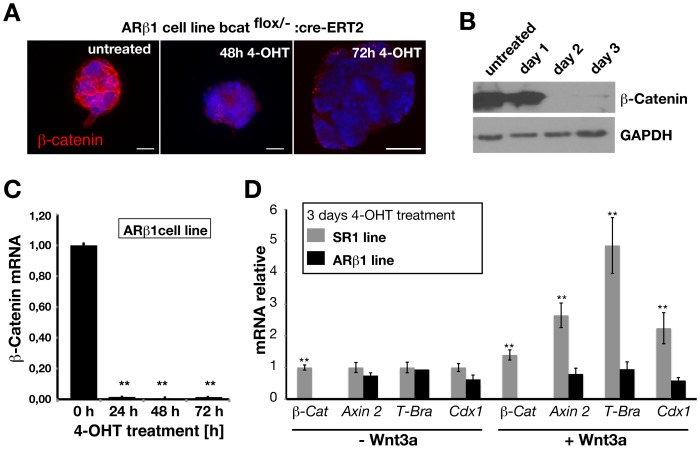
Treatment of ARβ1 *(βcat* ^del/flox^ :Cre-ER-T2) ES cells with 4-OHT induces Cre-mediated recombination of the *β-catenin* genomic locus leading to ablation of β-catenin. (A) Immunocytochemical staining for β-catenin protein on cultured ARβ1 cells reveals loss of β-catenin protein already at day two and appears to be absent at day three. (B) Western-immunoblotting against β-catenin demonstrates the almost complete loss of protein within 3 days of culturing. (C) *β-catenin* mRNA levels drop already 24 h after 4-OHT administration as revealed by quantitative RT-PCR (n = 3). ARβ1 ES cells do not respond to Wnt3a mediated canonical Wnt signaling after treatment with 4-OHT. (D) SR1 (*βcat* ^del/flox)^) and ARβ1 ES cells were treated for three days with 4-OHT and Wnt3a protein to induce canonical Wnt-signaling. Quantitative RT-PCR analysis of Wnt-target genes revealed that in contrast to SR1 cells ARβ1 cells are do not up-regulate significantly *Axin 2*, *T-Brachyury* and Cdx 1 (n = 3). *P<0,05, **P<0,01; student’s t-test. Error bars = s.e.m.; scale bar 10 µm.

Treatment of ARβ1 cells with 4-OHT leads to a decrease of β-catenin protein levels already after 24 hours and appeared almost completely absent after 2–3 days as shown by immunocytochemistry and Western immunoblotting (Fig. 1A and B). Similar, but even more prominent *β-catenin* mRNA levels are already down 24 h after 4-OHT administration (Fig. 1C). Next, we tested whether 4-OHT treated ARβ1 ES cells are able to respond to Wnt3a an activator of the canonical β-catenin dependant Wnt signaling pathway (Fig. 1D). We found that ARβ1 cells that were treated with 4-OHT were not able to induce expression of the Wnt-target genes *Axin 2*, *T-bra and Cdx 1* in contrast to SR1 control cells three days after 4-OHT supplementation. Taken together, these results demonstrate that β-catenin can be efficiently ablated by 4-OHT administration in ARβ1 ES cells and that this system can be used to study the role of β-catenin in ES cells.

**Figure 2:**
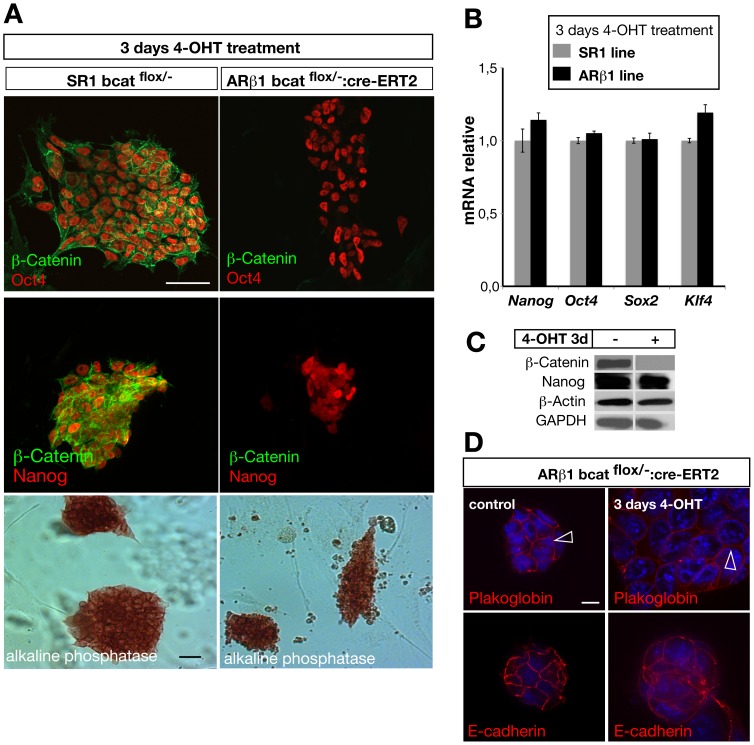
ARβ1 ES cells maintain expression of pluripotency markers at early time points after ablation of β-catenin. (A) Treatment with 4-OHT leads to loss of β-catenin protein in ARβ1 cells (right panel, green) but not in SR1 cells (left panel, green). The pluripotency markers Oct-4 and Nanog are present after ablation of β-catenin (right panel, red) demonstrated by immunocytochemical staining. Similar, ARβ1 cells still express alkaline phosphatase three days after ablation of β-catenin (visualized by enzymatic reaction using NBT/BCIP as substrate). (B) Quantitative RT-PCR analysis of the pluripotency genes *Nanog*, *Oct 4*, *Sox 2* and *Klf 4* reveal no statistically significant difference between SR1 and ARβ1 cells three days after treatment with 4-OHT (n = 3). (C) Western-immunoblotting demonstrating the loss of β-catenin in ARβ1 cells and the presence of Nanog protein after 4-OHT-treatment. (D) Ablation of β-catenin does not lead to a loss of cell-adhesion contacts. Staining for plakoglobin and E-Cadherin show localization to the cell membrane and cell-cell contacts (arrowheads). *P<0,05, **P<0,01; student’s t-test. Error bars = s.e.m.; scale bars: 50 µm (A), 10 µm (D).

To further characterize the ARβ1 ES cell line we analyzed next whether these cells show expression of pluripotency markers after β-catenin ablation. Immunostaining for Oct4 and Nanog on 4-OHT treated ARβ1 cells revealed the presence of these markers (Fig. 2A). In addition, they also exhibit alkaline phosphatase another marker for pluripotent ES cells (Fig. 2A). Similar results were obtained by analyzing the mRNA levels of *Oct4, Nanog*, *Sox2* and *Klf4* by performing quantitative real time PCR (Fig. 2B) and Western immunoblotting for Nanog (Fig. 2C). Our findings demonstrate that ES cells still express markers for pluripotency 3 days after the removal of β-catenin. Interestingly, we observed that β-catenin deficient ES cells retained their compact morphology although the colony size appeared smaller and exhibited localization of plakoglobin at the cell membrane (Fig. 2D). This finding might account for redundancy of plakoglobin and β-catenin in maintaining proper adhesion within the first three days after β-catenin ablation. (Fig. 2D). We also analyzed SR1 and ARβ1 ES cells after treatment with 4-OHT in long term cultures and observed no statistical significant differences in the expression of *Klf4*, *Nanog*, *Oct4* and *Sox 2* between recombined and non-recombined cells (Fig. S1).

### Ablation of β-catenin in Embryonic Stem Cells Leads to Cell Death

**Figure 3:**
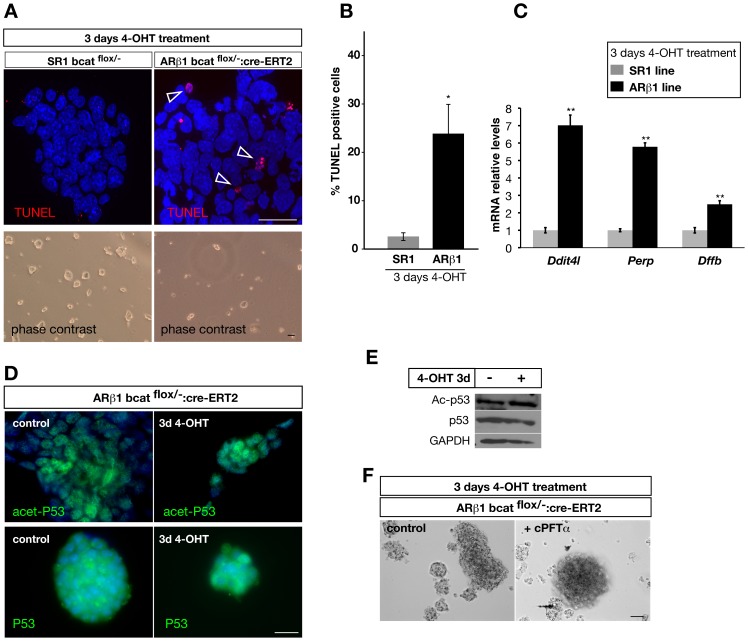
Removal of β-catenin in ARβ1 ES cells leads to an increase of apoptosis. (A) TUNEL staining on SR1 and ARβ1 ES cells after treatment with 4-OHT for three days shows that more cells undergo apoptosis in ARβ1 cells when β-catenin is absent (arrowhead). (B) Quantification of the number of TUNEL-positive cells after removal of β-catenin by 4-OHT treatment for three days. The number of cells was quantified by TUNEL staining followed by FACS sorting and revealed an average increase of 21% of apoptotic cells (n = 4). (C) The mRNA levels of the pro-apoptotic genes *Ddit4l*, *Perp* and *Dffb* were analyzed by quantitative RT-PCR in SR1 and ARβ1 ES cells after treatment with 4-OHT. In cells lacking β-catenin the mRNA levels were strongly and statistically significant up-regulated in comparison to the SR1 line (n = 3). (D) Immunocytchemical analysis of p53 and acetylated p53 protein in ARβ1 ES cells treated with 4-OHT for three days (right panel) or non-treated (control, left panel). In both conditions anti-p53 as well as anti-acetyl-p53 (Lys-382) staining was observed with similar cellular localization and intensity. (E) Western-Immunoblotting using anti-p53 and anti-acetyl-p53 (Lys-382) on lysates of ARβ1 ES cells treated with 4-OHT for three days to ablate β-catenin and non-treated cells. In both cell lysates similar amounts of p53 and acetylated p53 were observed. GAPDH protein levels were used to control for the loading amount. (F) The p53 inhibitor c-Pifithrin-α (cPFTα) was used to inhibit p53. ARβ1 ES cells were treated for three days with 4-OHT only (control) or together with cPFTα. Under both conditions a similar fraction of cells started to detach and to undergo cell death as shown under brightfield conditions. *P<0,05, **P<0,01; student’s t-test. Error bars = s.e.m.; scale bars: 25 µm (fluorescence pictures), 50 µm (transmitted light pictures).

When analyzing the early cellular events in embryonic stem cells after genetic removal of β-catenin we observed that many ES cells underwent cell death within the first days. ES cell colonies appeared much smaller and many cells detached from the colonies and the culture dish. (Fig. 3A, lower panel). Therefore, we performed TUNEL analysis to examine whether removal of β-catenin from ES cells turns on apoptosis as an early response. We observed that treatment of ARβ1 ES cells with 4-OHT results in a strong increase in TUNEL positive cells by approximately 20% (Fig. 3A and B).

To gain further insights into the transcriptional events after ablation of β-catenin in ES cells we performed gene array based transcriptome analysis combined with software based pathway analysis of ARβ1 and SR1 control cells treated for 3 days with 4-OHT. In accordance with the TUNEL analysis our experiments identified a group of pro-apoptotic genes involved in induction and regulation of cell death as being up-regulated. Among them, we selected three candidate genes, namely: *Ddit4l*, *Perp* and *Dffb* for further validation. Quantitative real time PCR analysis confirmed our gene array results. All three genes appear highly significant induced in ARβ1 ES cells after ablation of β-catenin by administration of 4-OHT (Fig. 3C) supporting our finding that ablation of β-catenin in ES cells leads to an onset of apoptosis as early cellular response. Additionally, our transcriptome analysis revealed that also genes involved in p53 signaling appeared to be strongly regulated. Therefore, we tested whether the observed cell death after ablation of β-catenin shows p53 dependence. Upon DNA damage or stress signals p53 activation includes an increase of p53 protein, relocation to the nucleus and several post-translational modifications such as acetylation and phosphorylation. Surprisingly, immunofluorescence analysis of 4-OHT treated and non-treated ARβ1 ES cells showed no differences in respect to nuclear re-localization or intensity of p53 and acetyl-p53 (Lys-382) (Fig. 3D). In concordance, we did not observe an up-regulation of p53 protein or an increase in lysine 382 acetylation of p53 shown by Western immunoblotting (Fig. 3E). Next, we treated ARβ1 ES cells with 4-OHT to ablate β-catenin and in addition with the p53 inhibitor c-Pifithrin-α (cPFTα). In accordance to our previous findings, we did not observe a rescue of the early cell death phenotype (Fig. 3F and data not shown). From these studies we conclude, that ablation of β-catenin in mouse ES cells results in the onset of apoptosis as an early cellular response, which is independent of p53.

### Ablation of β-catenin Affects Centrosome Function and Chromosome Stability

**Figure 4:**
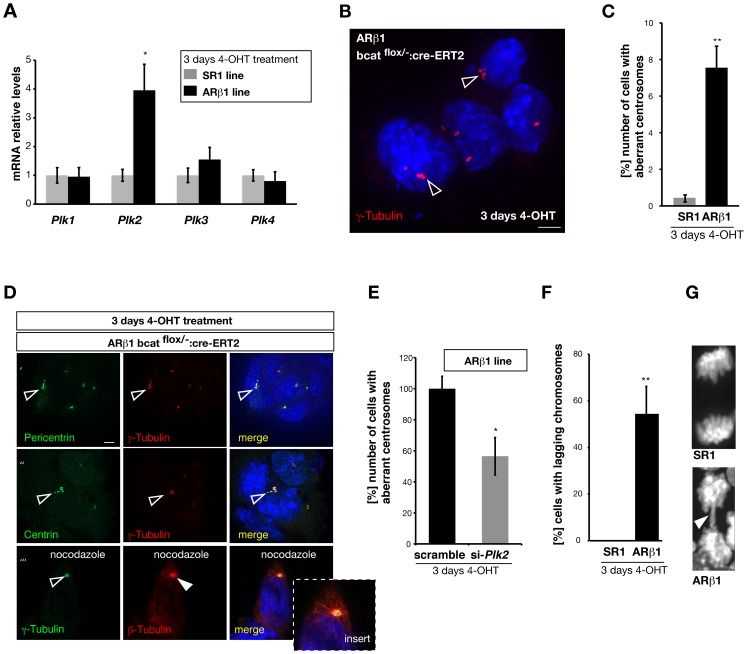
Depletion of β-catenin affects *Plk2* mRNA levels and centrosome number. (A) Quantitative RT-PCR analysis of the mRNA levels of the polo-like kinases 1–4 in SR1 and ARβ1 ES cells treated with 4-OHT. In β-catenin deficient cells *Plk 2* is up-regulated 4 fold whereas *Plk 1*, *3* and 4 are not statistically significant altered. (B) Immunocytochemical staining against γ-tubulin (red) in β-catenin deficient ES cells to label centrosomes. After ARβ1 cells were treated for three days with 4-OHT many ES exhibit more than two γ-tubulin-positive centrosomal structures arranged in clusters (open arrowheads). (C) Quantification of the appearance of cells with multiple (more than 2) γ-tubulin-positive structures SR1 and ARβ1 ES cells treated with 4-OHT. We found that about 8% of the β-catenin deficient ARβ1 cells exhibit multiple centrosomal structures compared to 0.2% in SR1 cells (n = 3). (D) Centrosome analysis in β-catenin deficient ARβ1 ES cells (following 4-OHT treatment for 3 days). Immunocytochemical staining show co-localization of both, Pericentrin (lane’) and Centrin (lane’’) with γ-tubulin (open arrowheads) on clustered centrosomal structures. To further validate for functional centrosomes β-catenin deficient ARβ1 cells were treated with Nocodazole (lane’’’). Staining for β-tubulin after cell recovery shows the reassembling of centrosome organized microtubules (arrowhead, red, open arrowhead indicates the γ-tubulin-positive centrosome, green). (E) ARβ1 ES cells treated with 4-OHT were transfected with si-RNA against *Plk 2* mRNA or control si-RNA (scrambled). β-catenin deficient ES treated with si-*Plk 2* show a reduction in the appearance of aberrant chromosomes namely multiple centrosomes (more than 2 and clustered) by 40% (n = 3). (F) β-catenin deficient ES-cells exhibit chromosomal lagging aberrations at early time-points after β-catenin ablation. Counting of the number of cells with lagging chromosomes after treatment for three days with 4-OHT revealed that about 50% of the mitotic ARβ1 ES cells exhibit this phenotype. Lagging chromosomes were absent in SR1 control cells (n = 3). (G) High magnification of SR1 control and ARβ1 nuclei after treatment with 4-OHT for three days. ARβ1 ES cells exhibit to a high degree lagging chromosomes (arrowhead) visualized with DAPI. *P<0,05, **P<0,01; student’s t-test. Error bars = s.e.m.; scale bars: 5 µm.

We realized when culturing ARβ1 ES cells in the presence of 4-OHT that the growth rate of cells deficient for β-catenin was strongly impaired compared to SR1 or non-treated ARβ1 cells (data not shown). This observation together with our data pointing to a cell death phenotype led us hypothesize that chromosome stability or centrosome function might be impaired after ablation of β-catenin in mouse ES cells. Therefore, we re-examined our gene array data and identified the *polo like kinases 2* and 3 genes (Plk2 and Plk3) as being strongly up-regulated. We performed real-time PCR analysis and could verify the strong and significant up-regulation of *Plk2* after β-catenin ablation in ARβ1 ES cells. *Plk3* appeared in the RT-PCR experiments only slightly up-regulated, however, not statistically significant (Fig. 4A). To confirm our findings we have additionally applied sh-RNA knockdown experiments of β-catenin in W4 wild type ES cell and analyzed the transcriptional levels of *Plk1-4* by RT-PCR and obtained similar results (data not shown). Since polo-like kinases are known to localize to centrosomes, are involved in centrosome duplication and are necessary to ensure genomic stability we addressed these issues next on our system.

We immunostained for centrosomes in β-catenin deficient ARβ1 ES cells by using an anti-γ-tubulin antibody and observed with high frequency cells with more than 2 centrosome-like structures (Fig. 4B). Interestingly, γ-tubulin-positive structures appeared always clustered (see arrowheads Fig. 4B). Quantification of this phenotype revealed an 17-fold increase in the appearance of multiple clustered centrosomes in β-catenin deficient ES cells compared to control cells three days after ablation (Fig. 4C). To exclude an effect caused by the presence of Cre-recombinase we generated a stable W4 wild type ES cell line carrying the same Cre-Er-T2 cassette as our SR1 line and did not find an effect on centrosome number after Cre induction (Fig. S2). To analyze whether the centrosomes in β-catenin deficient ES cells are functional, we stained first for Pericentrin and Centrin and could show co-immunolabeling with γ-tubulin (Fig. 4D, panel 1 and 2). Next, we applied Nocodazol wash-out experiments in order to examine the capability of the centrosomes in β-catenin deficient ES cells to nucleate microtubules. Co-staining with anti β- and γ-tubulin on ARβ1 ES cells treated for three days with 4-OHT followed by a nocodazol washout revealed indeed the potential of the clustered centrosomes to nucleate microtubules (Fig. 4D, panel 3 and insert). These data demonstrate that removal of β-catenin from ES cells leads to the formation of multiple clustered and functional centrosomes.

We have identified *Plk2* as being up-regulated after depletion of β-catenin. Since Plk2 is known to be involved in the regulation of centrosome duplication we wondered whether the appearance of multiple clustered centrosomes might be a consequence of this up-regulation. Therefore, we used a si-RNA approach to knock-down the mRNA levels of *Plk2* after ablation of β-catenin in ARβ1 ES cells and observed a reduction of approximately 40% of the appearance of multiple clustered centromeres (Fig. 4E). Based on this experiment we conclude that the up-regulation of *Plk2* might be one reason for the observed centrosome phenotype after ablation of β-catenin.

Centrosomes exhibit a pivotal role in spindle organization during cell division. Furthermore, other reports demonstrated that an aberrant number of centrosomes lead to chromosome segregation defects. Therefore, we analyzed β-catenin ablated ARβ1 ES cells for mitotic abnormalities and observed with high frequency the occurrence of lagging chromosomes during anaphase. Quantification revealed that 50% of the analyzed cells exhibited a lagging chromosome phenototype during anaphase (Fig. 4F and G). In accordance with this finding counting of the number of chromosomes in ARβ1 ES cells after β-catenin ablation or in W4 wild type ES cells after *β-catenin* down-regulation by sh-RNA revealed that more than 35% of the analyzed cells exhibited an abnormal set of chromosomes with more than 40 chromosomes.

Our data show, that the removal of β-catenin from ES cells affects the number of centrosomes and chromosomal stability at an early time-points. Furthermore, we provide evidence that the up-regulation of the *polo-like-kinase 2* might be involved in causing the observed phenotype.

## Discussion

The molecular and cellular functions of β-catenin in ES cells are in the focus of interest for many years. Its dual function in maintaining adhesive cell-cell connections which also encloses direct and indirect regulation of signaling properties and its important role as key signaling molecule of the Wnt pathway turned out to be essential characteristics of ES cells and their biology. This dual function however, the enormous impact of the Wnt signaling pathway on many cellular processes and the additional cellular functions of β-catenin outside of the classical Wnt- and adhesion pathway e.g. in telomerase or centrosome biology [35], [36] are very likely a major reasons why it turned out to be so difficult to narrow down its function in ES cells and why contradictory findings have been reported. Many studies have addressed the function of β-catenin in ES cells by using established or newly generated *β-catenin* null cells, which have been analyzed after a long culturing period. While analyzing established *β-catenin* null ES cells we realized that sometimes cell lines behaved unexpectedly different than wild-type ES cells in respect to their capacity to mediate Wnt-signaling when we re-introduced β-catenin by transfection with *β-catenin* expression vectors (A.R., unpublished observation and Ph.D. thesis A.R. University of Freiburg, Germany). Therefore, we have decided to generate new *β-catenin* mutant ES cell lines that would allow for Cre-mediated ablation of β-catenin by using the Cre-ER-T2 system *(β-cat^del/flox^:Cre-ER-T2*) to study its function [28]. In this approach only one allele has to recombine to achieve full ablation and furthermore, recombination can be induced at defined and controlled time-points by supplementation of the cell culture medium with 4-OHT. In other approaches *βcat* ^flox/flox^ or *βcat* ^del/flox^ alleles were used and recombination had to be achieved by viral infection to drive Cre-recombinase expression or by transfection of Cre expression plasmids [12], [27]. We found that in our ARβ1 ES cell line *β-catenin* mRNA is absent already 24 h after administration of 4-OHT. As expected, β-catenin protein decay is more delayed with protein levels being only slightly reduced after 24 h but almost completely absent after 48 h and 72 h. Importantly, we could show that recombined cells in contrast to non-recombined cells are not able to react on Wnt-signals by up-regulation of target genes. These data together with the expression of the pluripotency markers *Oct4*, *Sox2*, *Nanog*, *Klf4*, the presence of alkaline phosphatase and furthermore their cell morphology make us feel confident that our newly established cell line is a suitable tool to study β-catenin functions in ES cells. Importantly, the non-altered levels of expression of pluripotency markers 3 days after administration of 4-OHT are in agreement with results published by others that have used inducible recombination systems [12], [27]. Interestingly, Wnt signals are necessary to prevent the transition of ES cells to epiblast cells and some previously examined β-catenin deficient ES cell lines have been shown to express marker profiles reminiscent of epiblast cells [17], [24].

Along with the ablation of β-catenin we observed that many ES cell colonies became smaller and cells started to detach. TUNEL analysis revealed that β-catenin ablated ES cells undergo apoptosis. It has been reported that neuronal stem cells in the developing embryonic forebrain undergo apoptosis after the loss of β-catenin as a consequence of the loss of N-cadherin mediated adhesion [37], [38]. We observed that after the ablation of β-catenin E-cadherin and plakoglobin are localized at the cell membrane of the ES cells, which is in agreement with observations by others indicating that plakoglobin might compensate in ES cells for the loss of β-catenin in mediating cell adhesion [12], [26], [27]. However, β-catenin null ES cell lines have been established previously and also we observed that not all cells undergo apoptosis. Therefore, we conclude that these ES cell clones might have escaped from cell death which have up-regulated plakoglobin or other compensatory mechanisms. This is also in line with our observation that cell cycle speed in β-catenin deficient cells is reduced at early time-points after ablation and recovers to normal speed 8 to 10 days later (data not shown). We attempted to identify genes differentially regulated in β-catenin deficient ES cells by gene array combined with software based signaling pathway analysis. Here, we identified a group of genes, which have been described for having pro-apoptotic features or as being regulated by the p53-signaling pathway supporting our finding that ES cells undergo apoptosis shortly after depletion of β-catenin [39], [40], [41], [42], [43], [44], [45], [46]. However, it appeared that we could not detect alterations in the expression, re-localization or modification of p53 itself suggesting that the observed cell death is most likely p53 independent. This is in agreement with previous findings in wild-type ES cells [47], [48]. It could well be, that the observed cell-death phenotype is also in our ES cells due to inappropriate cell-adhesion features and that only those cells/colonies survived that have compensated for this. However, silencing of β-catenin in certain cancer cells has also been shown to induce apoptosis [42], [49], [50]. If any of these mechanisms might also account in our system needs to be clarified in more detail in future. Nevertheless, we have confirmed our set of data with a second approach by using a sh-RNA mediated knock-down (data not shown) to exclude cell death inducing effects due to over-expression of Cre-recombinase [51].

Our gene array analysis revealed also a strong up-regulation of the *polo-like-kinase 2* a major regulator of centrosome duplication [52]. Therefore, we analyzed the centrosomes in our ARβ1 ES cell line shortly after β-catenin ablation. Interestingly, we found to a high degree multiple centrosomes within the ES cells, which additionally appeared clustered. β-catenin has been shown previously to be required for centrosome amplification [53]. In this report a gain of function approach by over-expression of stabilized β-catenin in MDCK cells has been used leading also to the formation of extra-centrosamal structures. It is interesting to note that either too much or too little β-catenin results in the formation of extra centrosomes suggesting that the level of β-catenin has to be tightly controlled to ensure correct centrosomal duplication. However, important differences in the two approaches can be observed. β-catenin over-expression leads to extra centrosomal-like, γ-tubulin positive punctae that are rather sparse within the cell, lack pericentrin and are not functional. In contrast to this, we observed that the removal of β-catenin leads to the generation of clustered extra-centrosomes, which are pericentrin positive and importantly, functional in respect to nucleation of centrosome organized microtubules. We wondered whether the direct loss of β-catenin or the up-regulation of *Plk2* might have accounted for the appearance of extra-centrosome-like structures and addressed this question by si-RNA knock-downs of Plk2 in β-catenin deficient ES cells and observed a reduction in the number of cells exhibiting extra centrosomes. This might hint to an indirect role of β-catenin in the formation of ectopic centrosomes by up-regulation of Plk2 due to the loss of β-catenin although also other mechanisms can be assumed. The presence of extra-centrosomes leads often to chromosome segregation defects and chromosomal instability a phenomenon often observed in cancer cells [54], [55]. We observed in 50% of our β-catenin deficient cells a lagging chromosome phenotype, which could be the result of the presence of extra-centrosomes [55]. Whether this phenotype might account for our observation of an increase in cell death and the appearance of aberrant chromosome numbers is not clear and needs further investigation.

We aimed to study the early events after ablation of β-catenin and could show that the loss of β-catenin does not lead to a loss of pluripotency markers within the first 72 hours. This is an important finding in respect to the discussions regarding the role of β-catenin in maintaining pluripotency by analyzing long term cultured mutant ES cells. Interestingly, we did not find statistical significant alterations in the expression of *Klf4*, *Nanog*, *Oct4* and *Sox2* two month after ablation of β-catenin. Nevertheless, our findings point to adaption and selection processes resulting in alteration of expression of pluripotency markers in long term cultured β-catenin null ES cells. However, our study also demonstrates the important impact of β-catenin on key processes such as centrosome function and chromosome segregation within ES cells. These data, together with previously published studies on the role of β-catenin in ES cells show that it is difficult to analyze straight the action of β-catenin in ES cells and demonstrates the pitfalls long term cultured mutant ES cells can exhibit due to the sensitivity of the system, their ability for compensation and the complexity of action of the β-catenin signaling pathways.

## Supporting Information

Figure S1 **Analysis of pluripotency markers in long-term cultured mutant ES cells.** ARβ1 and SR1 ES cells were cultured for 2 month after 4-OHT induced ablation of β-catenin and analyzed by quantitative RT-PCR for the expression of pluripotency associated genes. The pluripotency genes *Nanog*, *Oct 4*, *Sox 2* and *Klf 4* reveal no statistically significant difference between SR1 and ARβ1 cells two month after treatment with 4-OHT. All PCRs were done in triplicates and repeated three times. All values are normalized to the corresponding SR1 cell values. Error bars = s.e.m.
(TIF)

Figure S2 **Analysis of** ***Cre*** **expression levels in mutant and wt ES cells.** (A) Comparison of the *Cre* mRNA expression levels in SR1 and stably transfected wild type W4 ES cells carrying a *Cre-ER-T2* cassette. Quantitative RT-PCR analysis revealed that both cell lines exhibit similar levels of *Cre* mRNA (n = 3 for SR1 and n = 5 for W4 cells). (B) To analyze whether the presence of Cre-recombinase has an effect on the number of centrosomes we analyzed W4 *Cre-ER-T2* cells after treatment with 4-OHT. No significant increase in centrosome number was observed after 4-OHT mediated induction of Cre expression. Error bars = s.e.m.
(TIF)
