# Supplementary figures and images for: Beta-Catenin Is Vital for the Integrity of Mouse Embryonic Stem Cells
Source: PLoS One. 2014 Jan 21;9(1):e86691. doi: 10.1371/journal.pone.0086691 (PMC3897734; doi:10.1371/journal.pone.0086691)

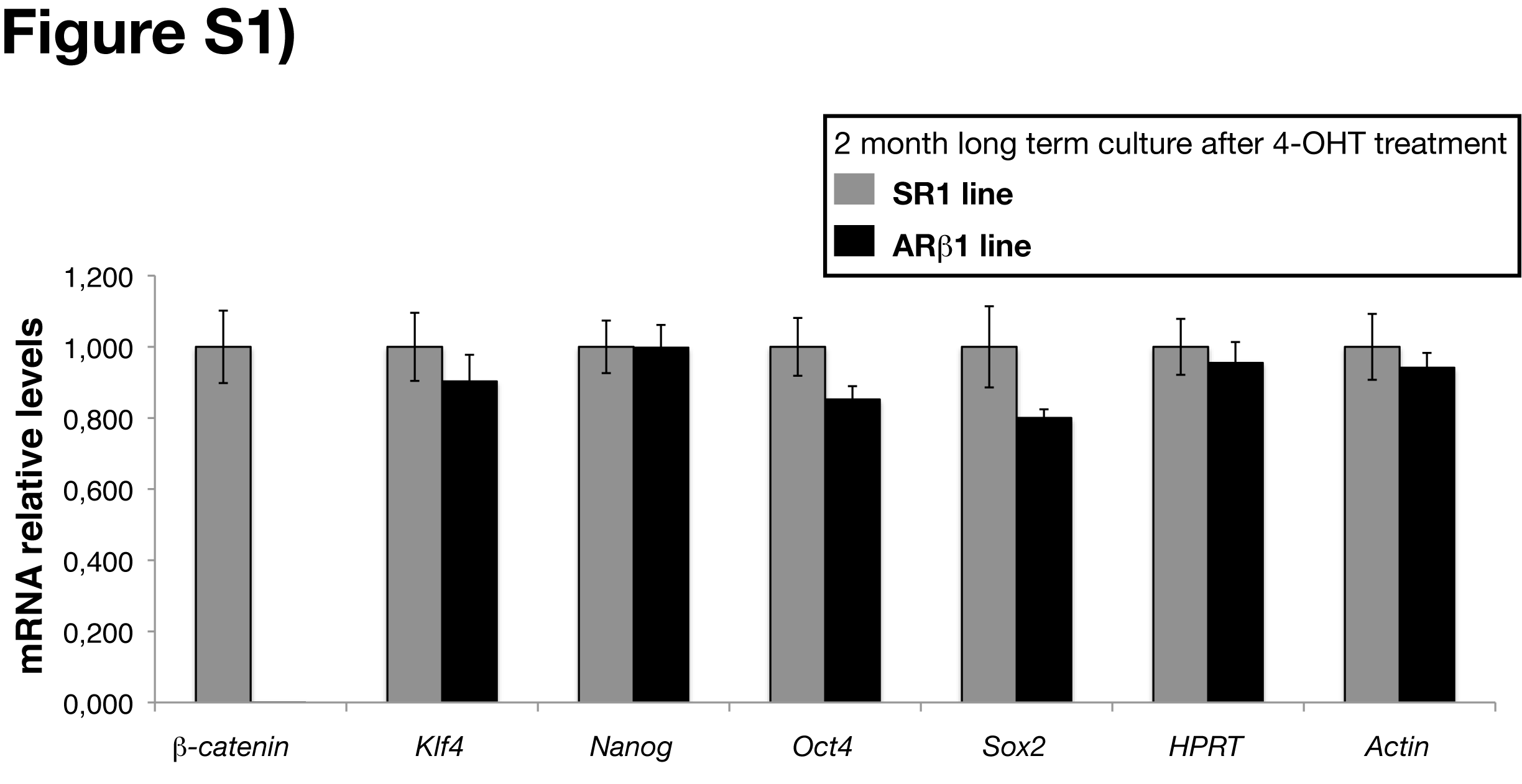

Supplement: Figure S1 — Analysis of pluripotency markers in long-term cultured mutant ES cells. ARβ1 and SR1 ES cells were cultured for 2 month after 4-OHT induced ablation of β-catenin and analyzed by quantitative RT-PCR for the expression of pluripotency associated genes. The pluripotency genes Nanog, Oct 4, Sox 2 and Klf 4 reveal no statistically significant difference between SR1 and ARβ1 cells two month after treatment with 4-OHT. All PCRs were done in triplicates and repeated three times. All values are normalized to the corresponding SR1 cell values. Error bars = s.e.m. (TIF) [file pone.0086691.s001.tif]

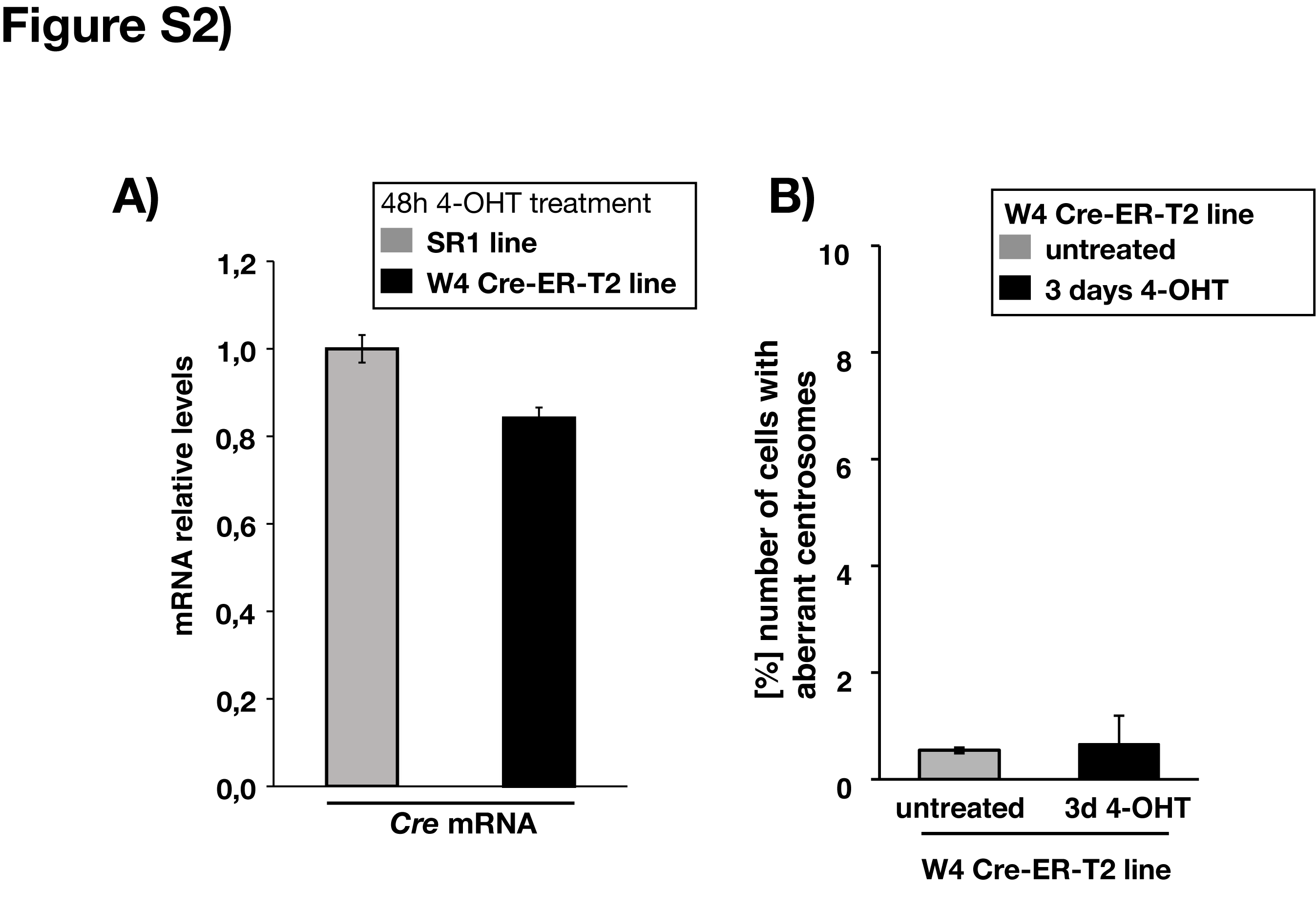

Supplement: Figure S2 — Analysis of Cre expression levels in mutant and wt ES cells. (A) Comparison of the Cre mRNA expression levels in SR1 and stably transfected wild type W4 ES cells carrying a Cre-ER-T2 cassette. Quantitative RT-PCR analysis revealed that both cell lines exhibit similar levels of Cre mRNA (n = 3 for SR1 and n = 5 for W4 cells). (B) To analyze whether the presence of Cre-recombinase has an effect on the number of centrosomes we analyzed W4 Cre-ER-T2 cells after treatment with 4-OHT. No significant increase in centrosome number was observed after 4-OHT mediated induction of Cre expression. Error bars = s.e.m. (TIF) [file pone.0086691.s002.tif]
